# GLINT: GlucoCEST in neoplastic tumors at 3 T—clinical results of GlucoCEST in gliomas

**DOI:** 10.1007/s10334-021-00982-5

**Published:** 2021-12-10

**Authors:** Benjamin Bender, Kai Herz, Anagha Deshmane, Vivien Richter, Ghazaleh Tabatabai, Jens Schittenhelm, Marco Skardelly, Klaus Scheffler, Ulrike Ernemann, Mina Kim, Xavier Golay, Moritz Zaiss, Tobias Lindig

**Affiliations:** 1 Department of Diagnostic and Interventional Neuroradiology, University Hospital Tübingen, Hoppe-Seyler-Str. 3, 72076 Tübingen, Germany; 2 Magnetic Resonance Center, Max Planck Institute for Biological Cybernetics, Tübingen, Germany; 3 Department of Biomedical Magnetic Resonance, University Hospital Tübingen, Tübingen, Germany; 4 Department of Neurology and Interdisciplinary Neuro-Oncology, University Hospital Tübingen, Tübingen, Germany; 5 Hertie Institute for Clinical Brain Research, Eberhard Karls University Tübingen, Tübingen, Germany; 6 Center for Neuro-Oncology, Comprehensive Cancer Center Tübingen-Stuttgart, University Hospital Tübingen, Eberhard Karls University Tübingen, Tübingen, Germany; 7 Cluster of Excellence iFIT (EXC 2180) “Image Guided and Functionally Instructed Tumor Therapies”, Eberhard Karls University Tübingen, Tübingen, Germany; 8 German Consortium for Translational Cancer Research (DKTK), Partner Site Tübingen, German Cancer Research Center (DKFZ), Tübingen, Germany; 9 Department of Neuropathology, University Hospital Tübingen, Tübingen, Germany; 10 Department of Neurosurgery, University Hospital Tübingen, Tübingen, Germany; 11 Department of Neurosurgery, Klinikum am Steinenberg, Reutlingen, Germany; 12 Institute of Neurology, University College London, London, UK; 13 Department of Neuroradiology, University Hospital Erlangen, Friedrich-Alexander University Erlangen-Nürnberg (FAU), Erlangen, Germany

**Keywords:** glucoCEST, Dynamic glucose enhancement, Glioblastoma, Chemical exchange saturation transfer, CEST

## Abstract

**Objective:**

Clinical relevance of dynamic glucose enhanced (DGE) chemical exchange saturation transfer (CEST) imaging has mostly been demonstrated at ultra-high field (UHF) due to low effect size. Results of a cohort study at clinical field strength are shown herein.

**Materials and methods:**

Motion and field inhomogeneity corrected T1ρ‐based DGE (DGE⍴) images were acquired before, during and after a d-glucose injection with 6.3 s temporal resolution to detect accumulation in the brain. Six glioma patients with clear blood–brain barrier (BBB) leakage, two glioma patients with suspected BBB leakage, and three glioma patients without BBB leakage were scanned at 3 T.

**Results:**

In high-grade gliomas with BBB leakage, d-glucose uptake could be detected in the gadolinium (Gd) enhancing region as well as in the tumor necrosis with a maximum increase of ∆DGE⍴ around 0.25%, whereas unaffected white matter did not show any significant DGE⍴ increase. Glioma patients without Gd enhancement showed no detectable DGE⍴ effect within the tumor.

**Conclusion:**

First application of DGE⍴ in a patient cohort shows an association between BBB leakage and DGE signal irrespective of the tumor grade. This indicates that glucoCEST corresponds more to the disruptions of BBB with Gd uptake than to the molecular tumor profile or tumor grading.

**Supplementary Information:**

The online version contains supplementary material available at 10.1007/s10334-021-00982-5.

## Introduction

Chemical exchange saturation transfer (CEST) has become a promising tool, for the depiction of micro-environmental and metabolic information within the human brain by utilizing the chemical exchange between protons of protein/metabolites and the abundant water proton pool. Glucose exhibits a hydroxyl CEST effect and was identified early as a potential natural contrast agent to image d-glucose uptake and metabolism in tumors [1, 2]. After the first successful application in the animal brain tumor model [3], the method was quickly also applied in humans at ultra-high field strengths [4–9]. Due to the current problems of scanning patients at ultra-high field (UHF) strengths, and particularly its lack of availability, a translation to a clinical field strength of 3 Tesla (3 T) is needed to increase the method’s reach and ensure its broader clinical evaluation. At ultra-high field strengths of 7 T and above the CEST effect is stronger than at 3 T [10], and the frequency separation between resonances of the exchangeable groups and the water peak is larger, leading to an improvement in signal detection. While subject motion can cause a pseudo-CEST-effect [11], nonetheless, successful implementations of chemical exchange saturation transfer sequences at 3 T have been recently reported [12, 13].

The exact underlying mechanism for the glucoCEST contrast is still under debate. Potential influencing factors are the perfusion, the permeability of the blood–brain barrier, the uptake kinetics of glucose and the local (tumor) metabolism which leads through the Warburg effect to a decrease of pH in the interstitial space [2], but the effect and interaction of these factors in-vivo are not clear. In this prospective study, we use a previously optimized 3D chemical exchange saturation spinlock (CESL) sequence to measure T1ρ‐based dynamic d-glucose enhanced (DGE⍴) signal in a cohort of patients with brain tumors. We examined the effects associated with blood–brain barrier breakdown and molecular tumor type and grade.

## Materials and methods

### Patients

This prospective study was conducted between 2018 and 2020 after prior approval by the local institutional review board (Ethics committee of the Medical Faculty at the Eberhard-Karls University of Tübingen and University Hospital Tübingen). All patients gave their written informed consent prior to the measurements. A total of 11 patients with suspected primary brain tumors were included in the study. Besides the DGE⍴-weighted CESL sequence, routine MRI data were available for all patients, which consisted of T1-w, T2-w and fluid-attenuated inversion recovery weighted (FLAIR-w) images without contrast agent and a post-T1-w contrast scan. Blood–brain-barrier breakdown was assessed by the presence of gadolinium contrast agent uptake within the tumor. All but one patient underwent concurrent resection or biopsy. Demographics and clinical information of all patients are summarized in Table 1. The first three patients have been included in a previous preliminary evaluation [12].

**Table 1:** Demographics, tumor location, tumor type, molecular tumor profile and progression-free survival for all patients

ID	Gender, age	Tumor location	Tumor type	Molecular status	BBB break down	Recurrency/progressive disease (months)
1	M, 70	R parietal	GBM °IV	IDH1/2 WT, ATRX retention, MGMT non-methylated	Yes	21
2	F, 61	L parieto-temporal	GBM °IV	IDH1/2 WT, ATRX retention, MGMT non-methylated	No	3
3	F, 54	R temporal	Giant cell GBM °IV	IDH1/2 WT, ATRX retention, MGMT intermediate methylated	Yes	
4	F, 46	R Temporo-insular	Diffuse astro-cytoma °II	IDH1 pos, 1p/19q no LoH, ATRX retention	No	
5	F, 29	L frontal	Diffuse astro-cytoma °II	IDH1 pos, 1p/19q no LoH, ATRX loss	(Yes)	
6	M, 70	R temporo-insular-frontal	Anaplastic astro-cytoma °III	IDH1/2 WT, ATRX retention, MGMT methylated	No	11
7	F, 63	L insular	GBM °IV	IDH1/2 WT, ATRX retention, MGMT non-methylated	Yes	
8	F, 52	R occipital	GBM °IV	IDH1/2 WT, ATRX retention, MGMT non-methylated	Yes	15
9	F, 58	R parieto-insular	GBM °IV	IDH1/2 WT, ATRX retention, MGMT non-methylated	Yes	7
10	M, 69	L parietal	GBM °IV	IDH1/2 WT, ATRX retention, MGMT methylated	Yes	1
11	M, 43	L fronto-insular	LGG	N/A	(Yes)	

*BBB* blood–brain barrier (= contrast enhancement), *F* female, *GBM* glioblastoma multiforme, *IDH* isocitrate dehydrogenase, *L* left, *LGG* low-grade glioma, *LoH* loss of heterozygosity, *M* male, *MGMT* *O*-6-methylguanine-DNA-methyltransferase, *R* right, *WT* wild type, *(Yes)* faint contrast enhancement

### MRI measurements

All imaging was performed at a clinical 3 T scanner (Prisma; Siemens Healthcare, Erlangen, Germany) with a 64-channel Rx Head Coil for optimal SNR. We applied a previously 3 T-optimized DGE-weighted CESL gradient echo (GRE) 3D snapshot sequence [12]. In short, the protocol consists of repeated blocks with 4 s of relaxation, a saturation phase (adiabatic prepared SL pulse of 4 µT and 120 ms locking time) [12] and snapshot GRE readout (Deshmane et al. 2019). Images were acquired with a 2 × 2 mm^2^ in-plane resolution (FoV 180 × 220 mm, bandwidth 700 Hz/px), with 12 slices (5 mm slice thickness), flip angle of 6° and parallel imaging acceleration factor 2 in phase encoding direction with GRAPPA reconstruction performed offline. The dynamic measurement included an unsaturated M0 scan at the beginning, followed by 32 measurements at each of five different frequency offsets (− 300, 0.6, 0.9, 1.2, and 1.5 ppm). All frequency offsets were measured after each other, resulting in 32 groups of 5 measurements each and a total of 161 measurements, including an M0 dummy scan at the beginning with a total scan time of 16:45 min, a temporal resolution of 6.3 s per scan and 31 s per group of offset measurements. If the measurement was part of a clinical scan, a contrast agent was applied after the DGE-weighted sequences.

### Glucose injection protocol

Prior to d-glucose infusions blood levels of potassium and sodium were checked and a baseline glucose level was measured to verify the safety of d-glucose injection (< 160 mg/dl). Patients were asked to fasten for 12 h, but some patients did not adhere to this request. A 20G or 21G peripheral venous catheter (PVC) was placed in the antecubital fossa and correct placement was tested with a 10 ml 0.9% saline flush. The patient was placed within the scanner, and a flushed 2 m perfusion line was attached to the PVC. After a baseline measurement of 3 min a d-glucose bolus of 1 ml clinical D20 glucose injection solution (20 g dextrose in 100 ml) per kilogram body weight (e.g. 70 ml, equal to 14 g dextrose for a patient with 70 kg body weight) was injected manually over a time period of 2–2.5 min and the perfusion line was flushed afterwards with 0.9% saline. At the end of the MR measurement blood glucose levels were measured again for safety reasons to rule out induced relevant hyperglycemia or reactive hypoglycemia.

### Postprocessing

Postprocessing was performed as in Ref. [12] and consisted of a retrospective rigid body motion correction using *elastix *[14], a dynamic B0 correction [15] using the phase information of the GRE readout and a *Z*-image calculation. *Z*-images were normalized dynamically by the corresponding measurement at − 300 ppm. For the B0 correction, *Z*-values off a subsequent group of offsets (0.6, 0.9, 1.2 and 1.5) were linearly interpolated voxel-wise. Afterwards, ∆DGEρ difference maps to baseline were calculated at each time-point and offset, using the mean of the first six images per offset as a baseline and the resulting DGEρ signal was filtered in time dimension by a box filter to get a more stable signal [12]. For visualization, the ∆DGEρ maps were filtered spatially, again using a box filter.

### Evaluation

All resulting images were rated visually for the presence of obvious motion artifacts. Patients with extreme motion were excluded from the following analysis. Presence of signal change after d-glucose injection, the start of signal change *t*_(start)_ after injection, time to maximum *t*_(max)_ after injection, and maximum signal change ΔDGEρ_(max)_ were evaluated for all patients. To reduce noise, mean ΔDGEρ values from 8 to 10 min post-injection were calculated as well. Spatial correlation between contrast agent uptake and DGEρ signal was evaluated visually by two neuroradiologists (BB, TL) in a consensus approach.

First and the last slice of the image stack were discarded, after which regions of interest (ROIs) were drawn manually on at least three slices that cover the tumor in each patient by a neuroradiologist (TL). If a clear contrast enhancement of the tumor was seen, contrast-enhancing areas (CE-ROI) and (if present) central necrosis (necrosis-ROI) were identified separately. If no or only faint contrast enhancement was visible, the whole T2-hyperintense tumor (FLAIR-ROI) was chosen as a single ROI, excluding obvious regions of edema. In all patients, an additional ROI in normal-appearing white matter was drawn on the same slices (WM-ROI).

Statistical evaluation was conducted with IBM SPSS Statistics (Version 24.0). A statistical significance was expected for any *p* < 0.05 (corrected for multiple comparisons).

## Results

**Fig. 1:**
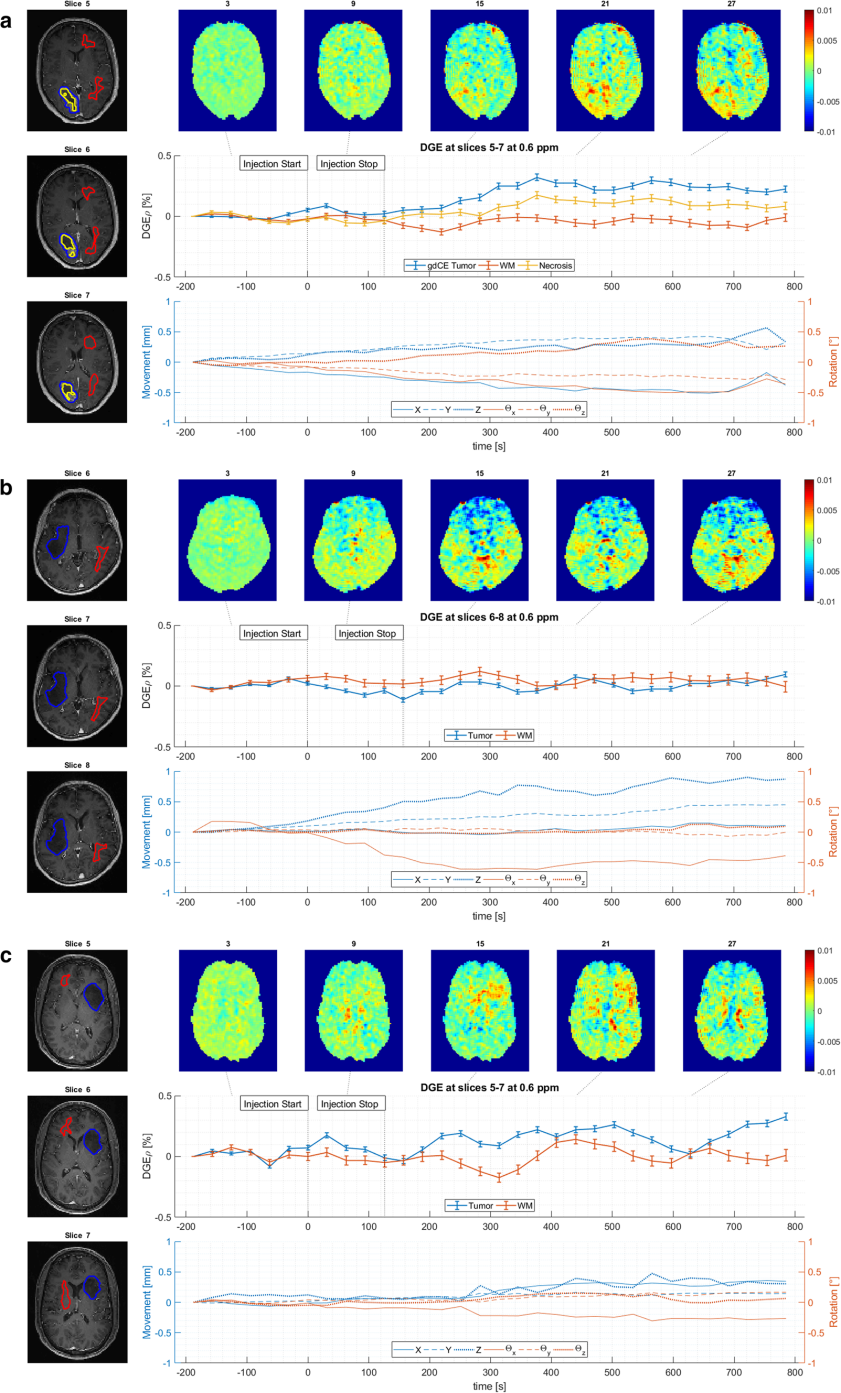
ΔDGEρ images, signal intensity profiles of the regions of interest and motion estimates for three typical examples are displayed, in a patient with clear contrast enhancement (**a**, patient no. 8), no contrast agent uptake (**b**, patient no. 4) and a faint contrast agent uptake (**c**, patient no. 11) of the tumor

**Fig. 2:**
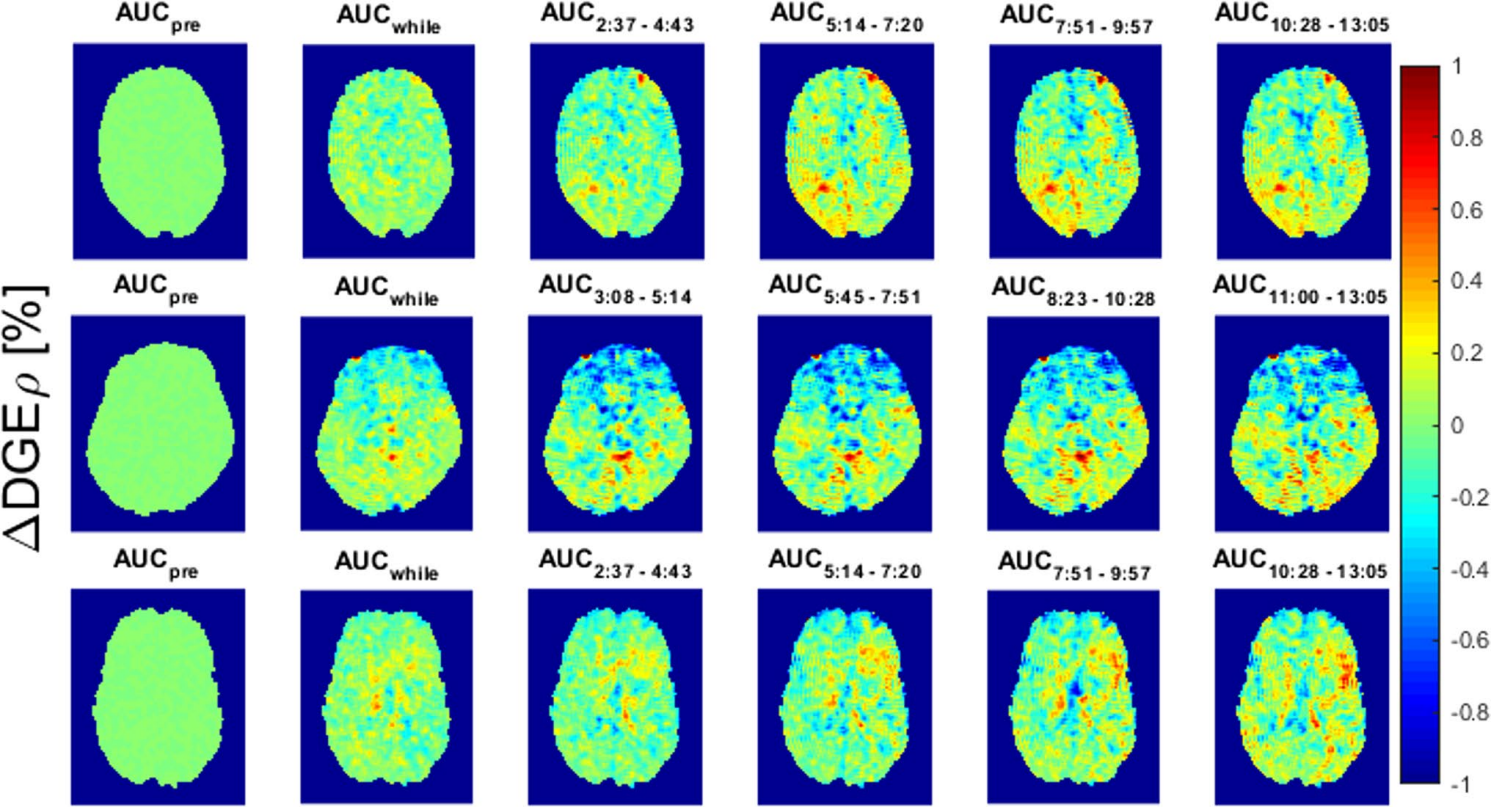
∆DGE⍴ AUC maps for patients 8 (1st row, slice 6). 4 (2nd row, slice 7) and 11 (3rd row, slice 6). The maps show the AUC during different timeframes: mean ∆DGE⍴ values before injection (1st column), during injection (2nd column) and after the injection (3rd-5th column). The time in the subscript indicates the timeframe after the beginning of the injection. Supplemental Fig. 1: ΔDGEρ at 8–10 min post-injection for each measured offset: each box plot consists of all voxels within the respective ROIs. ROIs of contrast-enhancing tumor, of tumor necrosis, of T2-hyperintense tumor without contrast enhancement, and in normal-appearing white matter were analyzed. Similar effects were measured at all offsets

Range and dynamics of motion differed between patients. Patients (no. 1, 7 and 9) with sudden shifts in the position showed more obvious motion artifacts than a constant small shift over time. In patient no. 1 movement was minimal until the late scans (around 10 min after d-glucose injection) where a sudden move was identified; therefore, evaluation was possible until this point of time. Patient no. 7 and 9 were excluded from statistical evaluation due to severe motion artifacts. No increase in DGEρ signal was seen in patients with no signs of a blood–brain barrier breakdown (no. 2, 4, and 6). In none of these patients was a pseudo-CEST contrast due to movement visible. Patients with a strong enhancement and necrosis (no. 1, 3, 8, 9 and 10), and also two patients (no. 5 and 11) with a faint enhancement showed a DGEρ signal increase starting approximately 4 min after injection with a maximum increase of ∆DGEρ between 0.2 and 0.4% after approximately 9 min, whereas tumor-unaffected white matter regions did not show any significant DGEρ increase (see Figs. 1, 2).

The distribution of the mean ΔDGEρ values at 8–10 min post injection for each group based on all voxels within the ROIs showed a normal distribution (see Supplemental Fig. 1). The effect did not differ between the offsets (see Supplemental Fig. 1), due to the broad DGEρ effect at this frequency range. We conducted a one-way ANOVA to assess the effect of tissue type (defined by ROIs) on mean ΔDGEρ at 8–10 min post injection: WM-ROI (*N* = 9, *M* = 0.00, SD = 0.46), FLAIR-ROI (*N* = 5, *M* = 0.12, SD = 0.18), CE-ROI (*N* = 5, *M* = 0.19, SD = 0.09) and necrosis-ROI (*N* = 4, *M* = 0.20, SD = 0.11). Leven’s test showed a violation of the assumption of homogeneity (*p* = 0.026). The mean ΔDGEρ at 8–10 min post injection differed significantly between ROIs, Welch’s *F*(3, 6.84) = 8.16, *p* = 0.012. Games–Howell post-hoc analysis revealed only a significant difference (*p* = 0.028) between WM-ROI and CE-ROI, with an increase in mean ΔDGEρ at 8 – 10 min post injection in the CE-ROI (0.19, 95% CI [0.04–0.34], but not for WM-ROI vs necrosis-ROI (*p* = 0.111; 0.20, 95% CI [− 0.07 to 0.41]) or FLAIR-ROI (*p* = 0.570; 0.11 95% CI [− 0.21 to 0.44]).

## Discussion

After the exclusion of two patients due to movement artifacts, a DGEρ effect could be shown at 3 T for 6/9 patients with a contrast-enhancing tumor, for which a mean signal increase at 8–10 min post injection was around 0.19% (95% CI 0.04–0.34%). This effect was smaller than a previous report at 7 T [5], which is in line with theoretical simulations [10], but the time curves nicely match previous published 3D results at 7 T that also included motion correction [6]. The measured effect was somewhat smaller than the results in three recent patients measured at 3 T with a mean signal increase at 2–7 min post injection of 0.52–0.97% [13], but the patients characteristics were not comparable. In addition, the dynamic B0 correction and normalization performed herein can also limit overestimations of effects [11, 12]. All patients within our study were treatment-naive, thus the findings are probably based on tumor-induced changes to perfusion, blood–brain barrier leakage, change in local pH and/or glucose metabolism. In glioblastomas (grade IV) cell densities and BBB leakage is expected to be higher, as in grade II and III tumors, where necrosis is absent. In the recent publication by Xu et al. post-treatment patients (surgery and probably radio-chemotherapy) were included. In such patients, the effect of therapy on the blood–brain barrier, local necrosis and inflammation can have significant effects on the local signal evolution. An interpretation and comparison of the results are, therefore, difficult.

From the different tumors and contrast agent uptake in our cohort, some careful hypothesis can be generated. It seems that the breakdown of the blood–brain barrier is the most important mediator of the DGEρ signal in-vivo. Perfusion changes often correlate with contrast enhancement in routine glioma imaging, although both imaging findings are based on different pathophysiologic changes, and thus local perfusion increase should be considered as one of the main drivers for the increase in DGEρ seen in our patient cohort. The kinetics seen in the DGEρ signal within the contrast-enhancing ROI do not show a typical perfusion related pattern, with a slow but steady increase over a longer period of time (Fig. 1a), which makes a large perfusion effect unlikely. Also, no DGEρ signal increase was detectable in histologically proven high-grade glioma with neither contrast enhancement nor signs of necrosis, but with increased proliferation rate and increased vascularity (no. 2). On the other hand, one patient with a proven diffuse astrocytoma (no necrosis, no increased vascularity, no. 5) and another with a typical imaging finding of a diffuse astrocytoma (no. 11), showed a positive DGEρ signal and a faint contrast enhancement, although necrosis should be absent and cell density lower than in grade IV tumors. From the results of the individual ROIs (see Table 2) the increase seen in FLAIR-ROIs was only based on the two patients with a faint contrast enhancement (see Table 3), while the other three patients showed similar values as for WM-ROI. The spatial effect was not strictly aligned to the contrast enhancement in all patients, which suggest a potential additional effect, like pH which is altered in many gliomas [16, 17]. Lower pH, as expected from extracellular lactate, leads to a lower hydroxyl exchange rate and thus better detectability via DGE [2], especially at lower clinical field strength.

**Table 2:** Mean ΔDGE at 8–10 min post injection within the individual ROI

Pat no	ΔDGE CE (%)	ΔDGE necrosis (%)	ΔDGE FLAIR (%)	ΔDGE WM (%)
1	0.25	0.16		0.05
2			− 0.02	0.00
3	0.20	0.14		− 0.01
4			− 0.01	0.06
5			0.41	− 0.06
6	0.04		0.03	− 0.05
7	− *0.04*	*0.01*		*0.01*
8	0.25	0.13		− 0.03
9	− *0.22*	− *0.08*		*0.05*
10	0.23	0.37		0.06
11			0.18	0.02

ROIs of contrast-enhancing tumor, of tumor necrosis, of T2-hyperintense tumor without or faint contrast enhancement, and in normal-appearing white matter were analyzed. Patient no. 7 and 9 were not included in the statistical analysis (marked in italic)

**Table 3:** Start of visible increase in ΔDGE signal after glucose injection, time to estimated maximum and maximum values in CE-ROI, maximum motion, result of visual inspection for contrast agent uptake (o = no, (+) = faint, +  = clearly defined) and blood glucose levels

ID	*t*_(start)_ in s	*t*_(max)_ in s	ΔDGE_(max)_ CE %	Max. motion	CE	Glucose start MR	Glucose end MR
1	15	288	0.32	1.5 mm/0.7°	++	111	158
2	N/A	N/A	N/A	0.3 mm/0.4°	O	93	134
3	15	288	0.3	0.5 mm/0.7°	++	154	201
4	N/A	N/A	N/A	0.9 mm/0.5°	O	94	140
5	15	416	0.51	0.5 mm/0.6°	(+)	83	124
6	N/A	N/A	N/A	0.3 mm/0.8°	O	103	98^a^
7	N/A	N/A	N/A	0.7 mm/0.6°	++	135	183
8	30	384	0.33	0.5 mm/0.5°	++	129	131^a^
9	N/A	N/A	N/A	2.0 mm/0.6°	++	129	152
10	15	512	0.24	0.6 mm/0.4°	++	100	150
11	160	320	0.22	0.5 mm/0.3°	(+)	104	99^a^

^a^Glucose levels after additional routine imaging, that lasted approx. 20 min

Motion artifacts are the main limitation at 3 T for DGE imaging, as the artificial effects on imaging can be much higher than the expected physiological changes. As artifacts arise mainly at the borders of tissues, such effects can spatially overlap with contrast-enhancing tumor and mimic physiologic findings [11]. Minimization of movement has therefore been identified as one of the most important tasks for successful DGE imaging at 3 T, and prolonged infusion times have been suggested [13]. To reduce sensations at the site of injection and to reduce the risk of a potential harmful thrombophlebitis we used an injection protocol that used (1) a reduced concentration of d-glucose (D20) in comparison to previous reports [5, 13, 18] and (2) a prolonged infusion time of 2–2.5 min in comparison to previous reports at 3 T and 7 T [5, 13, 18]. We could minimize motion by this approach, although in some patients motion > 0.5 mm or > 0.5° was detectable with concurrent signal changes even after motion correction and dynamic B0 correction.

Although our clinical results in patients with glioma at 3 T clearly demonstrate a strong association between DGEρ signal and blood–brain barrier disruption, further validation and evaluation are required to get a better understanding of the underlying physiological sources of signal formation in vivo. A combined effect of reduced pH within the extracellular space in high-grade gliomas and an increased concentration of d-glucose within the extracellular space when the blood–brain barrier is broken, could explain the findings. As the intracellular glucose concentration and its phosphorylated analogues are not detectable, due to the fast tumor metabolism, they should not contribute significantly to the signal formation [2]. For future measurements at 3 T, higher SNR, reduced motion (e.g. by using inflatable positioning pads) and additional measurement of pH changes, e.g. based on amine-weighted CEST imaging [19, 20] or 31-P spectroscopy [16], within the tumor and adjacent regions would be helpful to disentangle the influence of different physiologic parameters and artificial signal changes. A further increase in SNR and spatial resolution might be possible with the use of echo planar imaging (EPI) based sequences [21, 22], at the cost of potential signal loss in high-grade gliomas with hemorrhage. A whole-brain sequence using spatially non-selective pulses would also remove artifacts due to intensity differences in the slab profile and would allow improved motion correction of the brain. Nonetheless, our first clinical results associating DGEρ signal with Gd CE promisingly indicate that glucoCEST corresponds more to the disruptions of the blood–brain barrier with Gd uptake than to the molecular tumor profile or tumor grading.

For clinical protocols Gadolinum based imaging currently will remain the gold standard to identify BBB breakdown. To include DGEρ imaging within clinical protocols, shorter measurement times and higher SNR would be necessary. But it could be a potential method to avoid or reduce Gadolinium exposition in vulnerable patients in the future, if the association proofs to be strong.

## Supplementary Information

Below is the link to the electronic supplementary material.

Supplementary file1 (TIF 155 KB)
