# Supplementary figures and images for: GLINT: GlucoCEST in neoplastic tumors at 3 T—clinical results of GlucoCEST in gliomas
Source: MAGMA. 2021 Dec 10;35(1):77–85. doi: 10.1007/s10334-021-00982-5 (PMC8901469; doi:10.1007/s10334-021-00982-5)

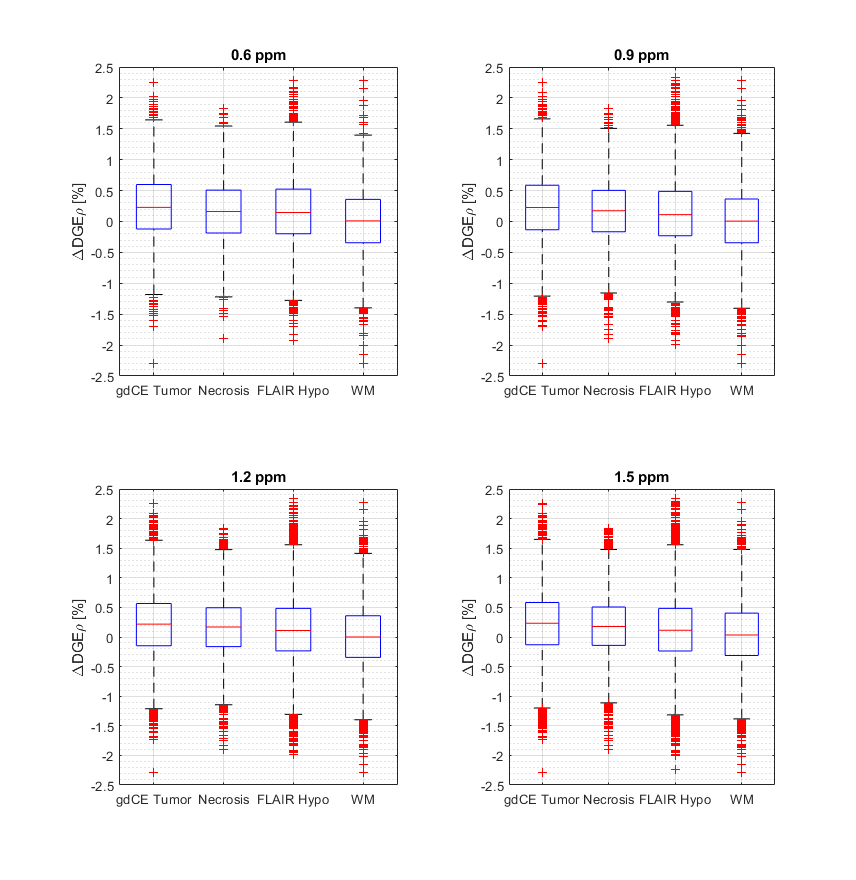

Supplement: Supplementary file 1 — Supplementary file1 (TIF 155 KB) [file 10334_2021_982_MOESM1_ESM.tif]
